# Viral heritability of neurocognitive performances in persons living with HIV and underlying viral residue variants

**DOI:** 10.1093/braincomms/fcag371

**Published:** 2026-10-05

**Authors:** Marius Zeeb, Mélanie Bieler-Aeschlimann, Paul Frischknecht, Kathrin Neumann, Lisa Jörimann, Jasmin Tschumi, Michael Huber, Katharina Kusejko, Enos Bernasconi, Helen Kovari, Karoline Leuzinger, Julia Notter, Matthieu Perreau, Cornelia Staehelin, Alban Ramette, Marcel Stöckle, Sabine Yerly, Alexandra Calmy, Karin J Metzner, Huldrych F Günthard, Matthias Cavassini, Roger D Kouyos, the NAMACO Study Group and the Swiss HIV Cohort Study, Irene A Abela, Karoline Aebi-Popp, Alexia Anagnostopoulos, Manuel Battegay, Enos Bernasconi, Dominique L Braun, Heiner C Bucher, Alexandra Calmy, Matthias Cavassini, Angela Ciuffi, Günter Dollenmaier, Matthias Egger, Luisa Elzi, Jan S Fehr, Jacques Fellay, Hansjakob Furrer, Christoph A Fux, Huldrych F Günthard, Anna Hachfeld, David H Haerry, Barbara Hasse, Hans H Hirsch, Matthias Hoffmann, Irene Hösli, Michael Huber, David Jackson-Perry, Christian R Kahlert, Olivia Keiser, Thomas Klimkait, Roger D Kouyos, Helen Kovari, Katharina Kusejko, Niklaus D Labhardt, Karoline Leuzinger, de T B Martinez, Catja Marzolini, Karin J Metzner, Nicolas Müller, Johannes Nemeth, Dunja Nicca, Julia Notter, Paolo Paioni, Giuseppe Pantaleo, Matthieu Perreau, Andri Rauch, Luisa P Salazar-Vizcaya, Patrick Schmid, Olivier Segeral, Roberto F Speck, Marcel Stöckle, Philip E Tarr, Alexandra Trkola, Gilles Wandeler, Maja Weisser, Sabine Yerly, Matthias Cavassini, Renaud Du Pasquier, Mélanie Métral, Samanta Simioni, Peter Brugger, Klemens Gutbrod, Andreas U Monsch, Ursi Kunze, Marianne Schneitter, Isaure Nadin, Severin Früh, Marc Schwind, Riccardo Pignatti, Stefanie Clarke, Frédéric Assal, Tobias Derfuss, Sebastian von Arx, Günter Eisele, Leonardo Sacco, Manuel Bertschi, Thomas Hundsberger, Alexandra Calmy, Thanh D Lecompte, Christoph Hauser, Alexia Cusini, Rainer Weber, Helen Kovari, Barbara Hasse, Philip Tarr, Marcel Stöckle, Christoph Fux, Enos Bernasconi, Caroline Di Benedetto, Alessandra Bruno, Patrick Schmid, Katharine Darling, Alexandra Scherrer, Katharina Kusejko, Sandra Chaudron, Yannick Vallet, Deolinda Alves, Isabella Locatelli, Bruno Ledergerber, Laurent Decosterd, Catia Marzolini, Cristina Granziera, Gunnar Krueger, Reto Meuli, Maria Vargas

**Affiliations:** Department of Infectious Diseases and Hospital Epidemiology, University Hospital Zurich, Zurich 8091, Switzerland; Institute of Medical Virology, University of Zurich, Zurich 8057, Switzerland; Division of Infectious Diseases and Leenaards Memory Centre, Lausanne University Hospital, University of Lausanne, Lausanne 1011, Switzerland; Department of Infectious Diseases and Hospital Epidemiology, University Hospital Zurich, Zurich 8091, Switzerland; Department of Infectious Diseases and Hospital Epidemiology, University Hospital Zurich, Zurich 8091, Switzerland; Department of Infectious Diseases and Hospital Epidemiology, University Hospital Zurich, Zurich 8091, Switzerland; Institute of Medical Virology, University of Zurich, Zurich 8057, Switzerland; Department of Infectious Diseases and Hospital Epidemiology, University Hospital Zurich, Zurich 8091, Switzerland; Institute of Medical Virology, University of Zurich, Zurich 8057, Switzerland; Institute of Medical Virology, University of Zurich, Zurich 8057, Switzerland; Department of Infectious Diseases and Hospital Epidemiology, University Hospital Zurich, Zurich 8091, Switzerland; Institute of Medical Virology, University of Zurich, Zurich 8057, Switzerland; Division of Infectious Diseases, Ente Ospedaliero Cantonale, Lugano 6500, Switzerland; Department of Biomedical Sciences, University of Geneva and University of Southern Switzerland, Lugano 6900, Switzerland; Center for Infectious Diseases, Klinik im Park, Zurich 8027, Switzerland; Clinical Virology, University Hospital Basel, Basel 4031, Switzerland; Division of Infectious Diseases and Hospital Epidemiology, Cantonal Hospital St. Gallen, St. Gallen 9007, Switzerland; Division of Immunology and Allergy, Lausanne University Hospital, University of Lausanne, Lausanne 1011, Switzerland; Department of Infectious Diseases, Inselspital, Bern University Hospital, University of Bern, Bern 3010, Switzerland; Institute for Infectious Diseases and Multidisciplinary Center for Infectious Diseases, University of Bern, Bern 3012, Switzerland; Division of Infectious Diseases and Hospital Epidemiology, University Hospital Basel, University Basel, Basel 4031, Switzerland; Laboratory of Virology and Division of Infectious Diseases, Geneva University Hospital, University of Geneva, Geneva 1205, Switzerland; HIV/AIDS Unit, Division of Infectious Diseases, University Hospital Geneva, University of Geneva, Geneva 1205, Switzerland; Faculty of Medicine, University of Geneva, Geneva 1205, Switzerland; Department of Infectious Diseases and Hospital Epidemiology, University Hospital Zurich, Zurich 8091, Switzerland; Institute of Medical Virology, University of Zurich, Zurich 8057, Switzerland; Department of Infectious Diseases and Hospital Epidemiology, University Hospital Zurich, Zurich 8091, Switzerland; Institute of Medical Virology, University of Zurich, Zurich 8057, Switzerland; Division of Infectious Diseases, Lausanne University Hospital, University of Lausanne, Lausanne 1011, Switzerland; Department of Infectious Diseases and Hospital Epidemiology, University Hospital Zurich, Zurich 8091, Switzerland; Institute of Medical Virology, University of Zurich, Zurich 8057, Switzerland

**Keywords:** HIV, neurocognitive domains, cohort studies, viral genome, neurocognitive impairment

## Abstract

Neurocognitive impairments (NCI) affect up to 50% of persons living with HIV (PLWH), even under anti-retroviral therapy. Evidence suggests that HIV-1 genotypes at least in part contribute to NCI, but most studies considered composite scores and did not examine specific neurocognitive domains. We considered neurocognitive domains assessed in the Neurocognitive Assessment in the Metabolic and Ageing Cohort, a sub study of the Swiss HIV Cohort Study (SHCS), and determined associations with HIV-1 residues, detected by full-length genome sequencing, previously linked to self-reported neurocognitive complaints (SRNC) in the SHCS, using linear regression. Additionally, we determined the heritability with Brownian motion- and mixed models based on viral *pol* phylogenies. In 192 out of 981 PLWH with full-length sequences, among the top residue variants identified, Env641E and Rev18I had been previously associated with SRNC. Respectively, of identified residue variants, point estimates correlated highly with previous SRNC-estimates from the SHCS for sensory skills (*r* = −0.71, *P* < 0.0001) and speed of processing (*r* = −0.71, *P* < 0.0001). Heritability estimates in 981 PLWH were significant for sensory skills [25.9%, 95% confidence interval (CI) 5.5–44.6%], motor skills (24.3%, CI 3.7–43.3%), and speed of processing (23.5%, CI 5.2–40.7%). Our findings support prior observations regarding SRNC-associated viral residues and confirm the relevance of the infecting HIV-1 strains for neurocognitive performances.

## Graphical Abstract

**Graphical Abstract:**
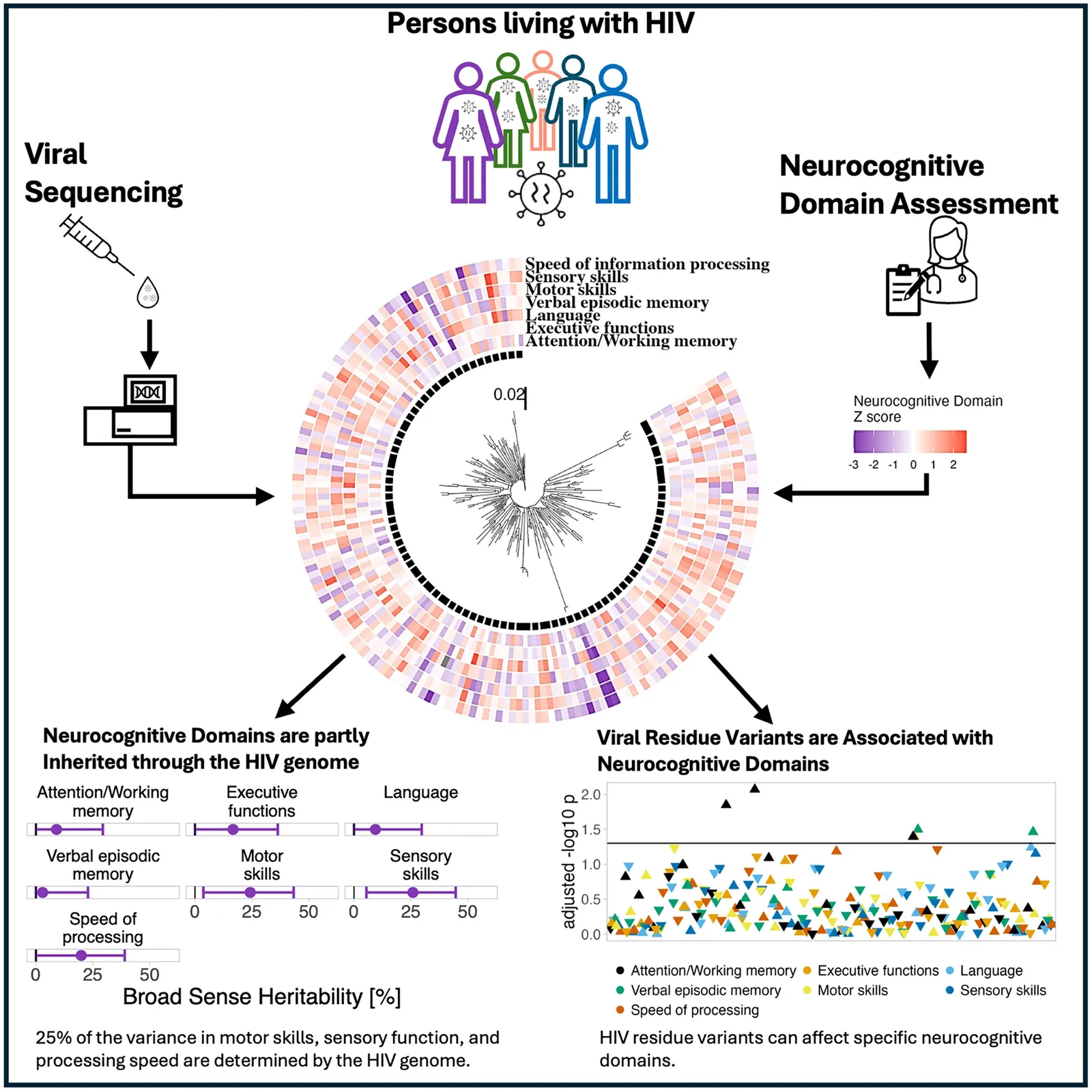

## Teaser

Zeeb *et al*. report that in persons living with HIV, viral gene variations, associated with self-reported neuropsychological complaints, are also associated with neurocognitive impairment in neurocognitive domains, i.e. motor and sensory skills. They conclude that further research may offer new avenues for preventive approaches to improve neurocognitive domains.

## Introduction

HIV-1 associated neurocognitive impairments (NCI) are a prevailing comorbidity in persons living with HIV-1 (PLWH). In Africa, a meta-analysis reported a prevalence of 46%, compared to 32% in a high-income setting such as Switzerland.^1,2^ While the introduction of effective anti-retroviral therapy (ART) did reduce the severity of the NCI impairments, from HIV-associated dementia to mild impairments, it remains a major health burden.^2-4^ The factors underlying its pathogenesis in the brain are complex and include HIV persistence, chronic inflammation, immune activation, metabolic changes, and adverse effects of ART.^5,6^ Noteworthy comorbidities, age, social and ethnic factors play important roles and must be considered, also as potential confounders in any analysis.^6^

The role of viral genomic variants is, however, only poorly understood. HIV infects the central nervous system (CNS) early after infection, particularly with CCR5 macrophage-tropic viruses via monocytes (the Trojan Horse theory), infection of blood–brain barrier (BBB) endothelial cells, or directly if the BBB is disrupted.^7,8^ Despite suppressive ART, viral RNA may remain detectable in the cerebrospinal fluid (CSF) and CNS, leading to the expression of viral pro-inflammatory proteins.^9-11^ The extent of this neuroinflammation depends on the genomic viral background and ongoing localized CNS evolution, distinct from plasma virus.^12,13^ Phenotypic differences were reported, for example, for subtype B Tat, which exhibits greater neuronal damage compared to subtype C Tat.^14,15^ Providing further evidence of viral genotype/residue variant involvement will elucidate underlying mechanisms of NCIs. This may guide precision medicine treatments for the management of comorbidities, e.g. treatments targeting neurotropic viruses. It might also add to the basic research of HIV cure approaches, a domain currently focusing on the HIV blood reservoir, but which ultimately needs to consider the brain as well. While multiple residue variants were reported in association with NCIs, this was mostly in untreated PLWH, with low CD4^+^ cell counts, and accordingly very severe neurocognitive outcomes (e.g. HIV-associated dementia).^16-18^ In contrast, current PLWH populations are characterized by much better immunological and virological status, but also higher ageing-related risk factors.^1,3^

We previously performed a viral genome-wide association study (GWAS) testing the association of viral residue variants with self-reported neurocognitive complaints (SRNC), consisting of three questions, proposed by the European AIDS Clinical Society in a population of PLWH with predominantly minor NCI forms.^1,3,16^ There, we observed only one significantly associated residue, but high viral heritability (explained phenotypic variance by the HIV-1 genome) estimates.^16^ As SRNC are rather unspecific, subjective, easily biased, and were shown to correlate only partially with HIV-associated neurocognitive disorder (HAND), the previously made observations necessitate validation using clinically validated phenotypes.^19^ While validation on HAND would be the obvious choice, and in line with most other viral genome studies, this generates additional issues.^17^ NCIs have diverse aetiologies, and reducing them to one composite score, such as HAND, may mask and oversimplify the underlying heterogeneity. A potential approach to overcome this issue and improve the capture of heterogeneous cognitive deficits is the validation of individual measures of different neurocognitive domains, for example, executive functions, language, motor, and sensory skills. Understanding the genomic determinants of specific domains is important for developing better treatments and prognostic tools for neurological health, particularly among ageing PLWH on ART.

Therefore, the present study aims to validate our prior findings and residues found by others using measures of neurocognitive domains as the outcome. These selected neurocognitive domains were known to be affected by HAND and were based on standardized neuropsychological tests as part of neuropsychological assessments of PLWH enrolled in the Neurocognitive Assessment in the Metabolic and Ageing Cohort (NAMACO) study, a sub-cohort of the Swiss HIV Cohort Study (SHCS).^3,20^ For our specific research objective, we determine whether known residue variants have consistent effects on neurocognitive domains.

## Materials and methods

### Study population

The study population consisted of PLWH enrolled in the SHCS and NAMACO study. The SHCS is a prospective, multicentric cohort study enrolling PLWH in Switzerland.^20^ NAMACO performed neuropsychological assessments in 981 PLWH. Inclusion criteria were ≥45 years old, enrolled in the SHCS, in care at one of seven cantonal university-affiliated hospital centres (Basel, Bern, Geneva, Lausanne, Lugano, St. Gallen and Zurich), and sufficient oral fluency in the local language for neuropsychological tests (no other inclusion/exclusion criteria applied).^1,3^ The SHCS is approved by the ethics committees of the participating institutions, and participants provided consent to the anonymized use of their data and specimens via the SHCS database and biobank for retrospective studies.

### Neurocognitive domains

Seven neurocognitive domains were assessed with neuropsychological tests (Table 1): verbal episodic memory, attention/working memory, speed of processing, executive functions, language, motor and sensory skills (including perception). The original NAMACO study selected these in line with the International Network for Strategic Initiatives in Global HIV Trials and Strategic Timing of Antiretroviral Treatment Study Group due to the clinically validated high sensitivity in detecting NCI, ease of administration, available standardization across ages and education groups and their availability in different languages.^3,21,22^ Specifically, the Hopkins Verbal Learning Test-Revised uses six parallel forms, designed to reduce practice effects of repeated longitudinal measurements, a known drawback of other tests.^23-25^ A 2-year waiting period between assessments additionally aimed to reduce the retest effect. Neuropsychological test results were converted to demographically adjusted standard scores (*z*-score). Neurocognitive domain-specific global *z*-scores (NDZS) were obtained by averaging domain-relevant test *z*-scores. An NDZS of zero indicates ‘normal’ skills, and one standard deviation below zero indicates ‘impaired’ skill in the respective neurocognitive domain according to the Frascati criterion.^26^

**Table 1:** Neurocognitive domains and underlying neuropsychological tests for their assessment

Neurocognitive domain	Neuropsychological test
Verbal episodic memory	Hopkins Verbal Learning Test-Revised, Total learning
	Hopkins Verbal Learning Test-Revised, Delayed recall
	Hopkins Verbal Learning Test-Revised, Retention
	Hopkins Verbal Learning Test-Revised, Recognition discrimination index
Attention/working memory	WAIS-IV Digit Span, Forward
	WAIS-IV Digit Span, Backward
Speed of processing	Colour Trails I
	WAIS-IV Digit Symbol subtest
Executive functions	Colour Trails II, Flexibility errors
	Colour Trails II, Interference index
	5-points Figural Fluency, Productivity
	5-points Figural Fluency, Perseveration
	Victoria Stroop Test (stroop-like errors/interference index)
	Category Fluency
Language	Victoria Stroop Test, Part D (Colour Naming)
	Category Fluency
Motor skills	Finger Tapping Test, Dominant
	Finger Tapping Test, Non-dominant
	Grooved Pegboard Test, Dominant
	Grooved Pegboard Test, Non-dominant
Sensory skills	Grooved Pegboard Test, Dominant
	Grooved Pegboard Test, Non-dominant
	Victoria Stroop Test, Part D (Colour Naming)

### Next-generation sequencing

Near full-length HIV-1 next-generation sequencing (NGS) for 192 out of all 981 PLWH was obtained either from plasma RNA or proviral DNA filtered for APOBEC-induced mutations.^27^ The preparation, amplification, and sequencing protocols are described by Giallonardo *et al*. and other publications.^28-32^ We obtained consensus whole genome sequences (WGS) using *de novo* assembly and aligned reads to the HXB2 reference sequence using SmaltAlign (https://github.com/medvir/SmaltAlign). Individual gene sequences from WGS were extracted locally with rBLAST 1.2.0 using HIV-1 subtype reference panels from the Los Alamos HIV sequence database.^33^ Gene codon alignments were obtained with MACSE 2.05.^34^ Sequences were processed with R 4.2.1 and PLINK v2.00a3.7.^35^ For each HIV-1 gene and PLWH, we considered the NGS sequence with the most coverage, allowing multiple source sequences per individual, but only one sequence per gene.

### Risk factors and confounders

Across analyses, we adjusted for the following risk factors and potential confounders, updated for each follow-up visit: sex; age; education (with/without additional education to mandatory school); HIV transmission mode [men-having-sex-with-men (MSM), heterosexual (HET), other]; ethnicity (White, non-White); CD4 T cell count history (AUC); HIV RNA load history (AUC); ever used antidepressants; ever reported depression; ever used recreational drugs; neurological disease/event [CNS infections, stroke, brain surgery, excluding HIV-1-associated dementia or -associated peripheral neuropathy (PNP)]; ever used efavirenz; prior HCV infection and prior HBV infection.

### Association analyses

In a previous viral GWAS study, we reported associations with SRNC.^16^ Here, we considered associated residues (*P* < 0.005) with any SRNC (Supplementary Table 1) and residues associated with HAND or CSF tropism reported by others (Supplementary Table 2). For the selected residues, we determined the respective residue effect sizes on NDZS in 192 PLWH with WGS. For the outcome, we calculated the area under the curve (AUC) of each NDZS over three follow-ups. We chose the AUC approach to define the cumulative neurocognitive burden while accounting for irregular visit intervals in NAMACO. Moreover, this approach is consistent with the previous study, which also used an AUC definition. We generated multiple sequence alignments per gene with MAFFT v.7.515 L-INSI, considered all residue variants with a frequency ≥10 (excluding all others) and performed linear regression analysis across all NDZS AUC outcomes and residues (population majority amino acid as reference).^36^ We addressed multiple testing with the Bonferroni correction. As this was an exploratory validation study, we did not conduct a formal power analysis. In addition to risk factors and potential confounders, we adjusted for HIV-1 genome population structure using principal components, calculated with Eigensoft 7.2.0.^37^ Finally, we determined the Pearson correlation between the effect estimates of residue-SRNC and residue-NDZS.

### Heritability

We determined broad-sense viral heritability of NDZS, including all 981 PLWH with either partial *pol* or near-full-length sequence, at the first NAMACO visit in three ways: (1) with mixed linear models in the R package lme4, using phylogenetic clusters as a random effect, based on distance-thresholds (0.06, 0.05, 0.04, 0.03), derived from partial *pol* genes. We determined the significance of heritability with a likelihood ratio test, comparing the model with cluster information to the model without (*P* significance threshold < 0.05). We obtained confidence intervals (CIs) with bootstrapping. (2) Brownian motion, which assumes random drift. (3) Ornstein–Uhlenbeck, which additionally accounts for selection pressure. We adjusted the analyses for risk factors and potential confounders. In the case of (1) directly, (2) and (3) indirectly, we did so by using residuals of an adjusted linear regression as the model outcome.^31^ (2) and (3) used the R package ‘poumm’.^38^ To account for HIV-1 subtype effects, we performed this analysis once with only subtype B and once with all subtypes. As an alternative to the AUC definition at one timepoint, we repeated the analysis with NDZS trajectories, defined as the linear slope over three visits. We inferred all underlying phylogenetic trees, including 1000 bootstraps, with IQ-Tree 2.2.0 using ModelFinder to select the substitution model.^39,40^

## Results

We included 981 PLWH with at least one NAMACO study follow-up; among those, 716 had two, and 551 had three visits. Across most neurocognitive domains, PLWH remained stable or improved over the observation period (Table 2).

**Table 2:** Population characteristics

	Visit 1	Visit 2	Visit 3
Characteristics^a^ at NAMACO follow-up	*N* = 981	*N* = 716	*N* = 551
Neurocognitive domain global *z*-scores			
Verbal episodic memory	0.11 (−0.44, 0.78)	0.22 (−0.44, 0.78)	0.22 (−0.44, 0.78)
Attention/working memory	−0.17 (−0.67, 0.34)	0.00 (−0.67, 0.50)	0.17 (−0.50, 0.67)
Speed of processing	−0.11 (−0.73, 0.37)	0.09 (−0.51, 0.56)	0.27 (−0.30, 0.74)
Executive functions	0.08 (−0.30, 0.43)	0.26 (−0.14, 0.58)	0.31 (−0.04, 0.63)
Language abilities	0.46 (−0.35, 1.11)	0.62 (−0.18, 1.21)	0.51 (−0.18, 1.10)
Motor skills	−0.24 (−0.75, 0.33)	−0.17 (−0.75, 0.33)	−0.17 (−0.75, 0.34)
Sensory skills	0.31 (−0.38, 0.87)	0.37 (−0.30, 0.93)	0.33 (−0.25, 0.91)
Birth year	1961 (1955, 1965)	1962 (1956, 1965)	1962 (1956, 1965)
Age [years]	53 (49, 59)	55 (52, 61)	57 (54, 62)
Sex [female]	197 (20%)	136 (19%)	108 (20%)
Higher education completed	849 (87%)	623 (87%)	486 (88%)
HIV transmission mode			
MSM	509 (52%)	402 (56%)	310 (56%)
HET	322 (33%)	212 (30%)	169 (31%)
Other	150 (15%)	102 (14%)	72 (13%)
Ethnicity non-White	81 (8%)	59 (8%)	46 (8%)
EFV use	562 (57%)	425 (59%)	331 (60%)
Antidepressant use	231 (24%)	192 (27%)	169 (31%)
Depression	346 (35%)	290 (40%)	257 (47%)
Recreational drug use	112 (11%)	90 (13%)	79 (14%)
Neurological disease/event	61 (6%)	62 (9%)	63 (11%)
Hep C	167 (17%)	116 (16%)	87 (16%)
Hep B	281 (29%)	210 (29%)	162 (29%)

^a^Median (interquartile range)/*n* (%).

### Top significant associations are similar between NDZS and SRNC

We assessed associations with 36 unique residue variants previously determined in a GWAS with SRNC.^16^ Of those, 14 were associated with concentration difficulties, 11 with frequent memory loss, 18 with cognitive slowing and 13 with the combination (Supplementary Table 1). We considered the NDZS AUC from 192 PLWH with three follow-ups and WGS. Between 59 and 63% of WGS originated from proviral DNA (Supplementary Table 3). The population characteristics were similar to those without WGS (Supplementary Table 4). Among the original 36 residue variants, 19 were present in the current study population (Supplementary Table 1). We chose an unbiased approach by determining the association of each residue variant with all NDZS, independent of the SRNC originally associated with the respective residue. The strongest associations were found for Env L641E with lower speed of processing (*P* = 0.0039), Rev L18I with increased sensory skills (*P* = 0.0042) and language abilities (*P* = 0.0057), and Tat T64N with increased sensory skills (*P* = 0.025). However, after multiple testing adjustment, none remained significant (Supplementary Fig. 1). Nevertheless, Env L641E was also the most significant residue variant found in association with SRNC, specifically increasing frequent memory loss. Tat T64N was the second most significant association, specifically with lower cognitive slowing.^16^

### Effect estimates of SRNC and NDZS correlate

Despite no significant residue variants, we observed strong and significant correlations between residue effect size estimates for SRNC and residue effect size estimates for NDZS (Fig. 1A). The Pearson correlation was largest for sensory skills (*r* = −0.71, *P* < 0.0001) and speed of processing (*r* = −0.71, *P* < 0.0001) and verbal episodic memory (*r* = −0.59, *P* = 0.0002) (Fig. 1A).

**Figure 1:**
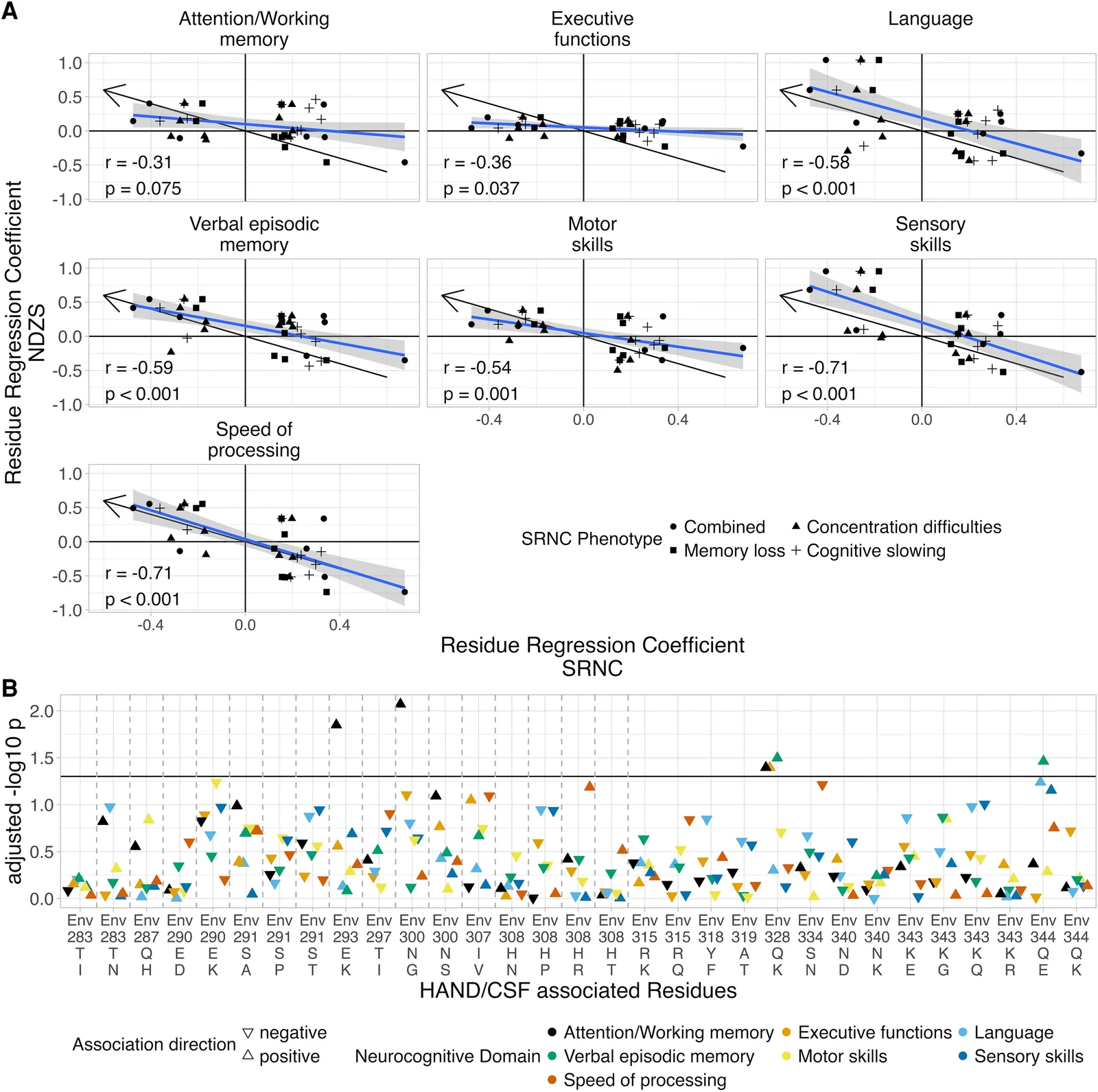
**Neurocognitive associations with the viral genome.** (**A**) Each dot represents a residue, the *x* axis corresponds to the effect estimate on the SRNC (indicated by the dot shape), the *y* axis corresponds to the effect estimate on the neurocognitive domain (indicated by the panel heading). *r* and corresponding *P* value are obtained from the Pearson correlation between the effect estimates (*N* = 34 per panel). The black arrow shows the theoretically perfect correlation between estimates, while its direction indicates which residues are associated with an improvement in both SRNC and neurocognitive domains. (**B**) Associations of residues with neurocognitive domains. Of those residues previously reported to be associated with HAND or CSF tropism.^17^ Each triangle represents a −log10 *P* value, corresponding to the association of the residue with the respective neurocognitive domain. The residue is indicated on the *x* axis, from the top: first the HIV gene, second the position, third the wildtype amino acid (used as a reference) and last the amino acid residue. Line at the top indicates the significance threshold. (**A**/**B**) Individual effect estimates for each residue, were obtained from multivariate linear regression (*N* = 192) adjusted for the following covariates: sex; age; education (with/without additional education to mandatory school); HIV transmission mode (MSM, HET, other); ethnicity (White, non-White); CD4 T cell count history (AUC); HIV RNA load history (AUC); ever used antidepressants; ever reported depression; ever used recreational drugs; neurological disease/event (CNS infections, stroke, brain surgery, excluding HIV-1-associated-dementia or -associated-PNP); ever used efavirenz; prior HCV infection; prior HBV infection. We adjusted for HIV-1 genome population structure using principal components. SRNC, self-reported neurocognitive complaints; CSF, cerebrospinal fluid; HAND, HIV-associated neurocognitive disorder; env, HIV envelope gene.

We determined associations between NDZS and residue variants previously linked to HAND or CSF tropism (Fig. 1B; Supplementary Table 2).^17^ Significantly associated were Env E293K (*P* = 0.014) and Env G300N (*P* = 0.0085), both with increased attention/working memory; Env Q328K, with increased verbal episodic memory (*P* = 0.032), attention/working memory (*P* = 0.04) and executive functions (*P* = 0.04); and Env Q344E, with increased verbal episodic memory (*P* = 0.034). Of note, the widely reported Env H308R showed a trend for increased speed of processing (*P* = 0.065) (Fig. 1B).

### NDZS show viral heritability

To infer the broad-sense viral heritability of each neurocognitive domain, we considered the NDZS at visit 1 in 981 PLWH. For the mixed models, we used phylogenetic clusters restricted to HIV-1 subtype B (Fig. 2A/C) and including all subtypes (Supplementary Fig. 2). Overall, we observed a trend of higher estimates with smaller distance-thresholds. The heritability estimates were significant for motor skills (24.3%, CI = 3.7%, 43.3%, *P* = 0.019, subtype B; 20.7%, CI = 2.3%, 38.3%, *P* = 0.026, all subtypes; distance-threshold = 0.03), sensory skills (25.9%, CI = 5.5%, 44.6%, *P* = 0.019, subtype B; 24.1%, CI = 5.7%, 41.1%, *P* = 0.016, all subtypes; distance-threshold = 0.03), speed of processing (23.5%, CI = 5.2%, 40.7%, *P* = 0.02, all subtypes; distance-threshold = 0.03), attention/working memory (15.8%, CI = 5.1%, 26.5%, *P* = 0.0014, subtype B; 13.2%, CI = 3.3%, 23.5%, *P* = 0.0035, all subtypes; distance-threshold = 0.05) and executive functions (11.4%, CI = 1.5%, 21.6%, *P* = 0.024, all subtypes; distance-threshold = 0.05) (Fig. 2). In contrast, the Brownian motion and Ornstein–Uhlenbeck models with partial *pol* or WGS showed no clear evidence for heritability, although they exhibited similar trends as the mixed model approach (Supplementary Figs 3 and 4).

**Figure 2:**
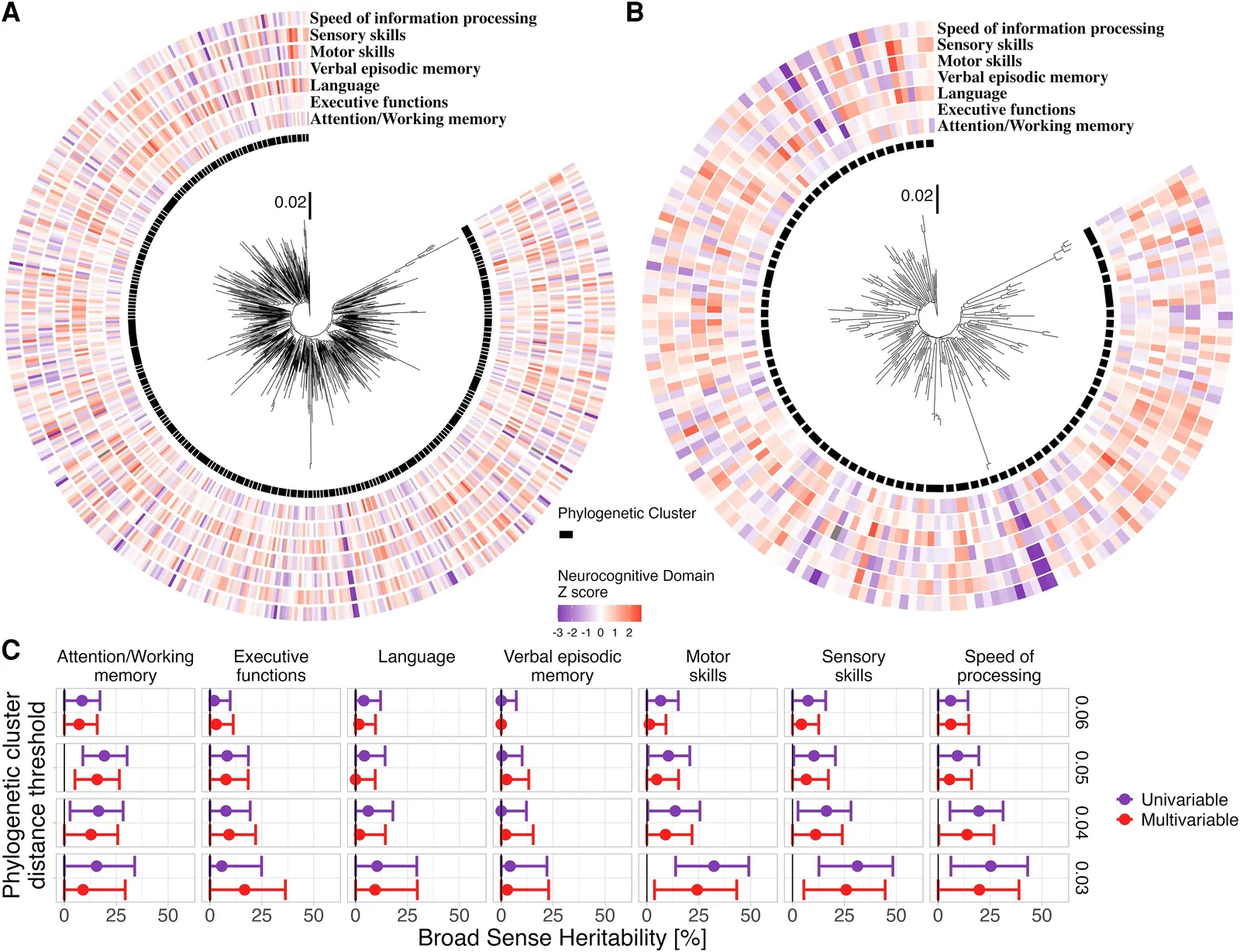
**Viral heritability for each neurocognitive domain.** (**A**/**B**) Phylogenetic trees based on partial pol sequences of subtype B, including clustering sequences using a distance of 0.06 (*N* = 533) (**A**) and 0.03 (*N* = 180) (**B**). Phylogenetic trees, including 1000 bootstraps, were inferred with IQ-Tree 2.2.0 using ModelFinder to select the substitution model. (**C**) Heritability estimates and their bootstrapped 95% CIs, inferred with linear mixed-effect models, incorporating phylogenetic clusters based on partial pol sequences (*N*_0.06_ = 533, *N*_0.05_ = 422, *N*_0.04_ = 313, *N*_0.03_ = 180) as random effects. Linear mixed-effect models were additionally adjusted for the following covariates: sex; age; education (with/without additional education to mandatory school); HIV transmission mode (MSM, HET, other); ethnicity (White, non-White); CD4 T cell count history (AUC); HIV RNA load history (AUC); ever used antidepressants; ever reported depression; ever used recreational drugs; neurological disease/event (CNS infections, stroke, brain surgery, excluding HIV-1-associated -dementia or -PNP); ever used efavirenz; prior HCV infection; prior HBV infection.

### Viral heritability over time

We determined the heritability of the NDZS evolution in 551 PLWH with three NAMACO visits. We found a significant heritability when restricting to subtype B for executive function (21.7%, CI = 4.7%, 37.1%, *P* = 0.017, distance-threshold = 0.05) and attention/working memory (17.5%, CI = 0.7%, 33%, *P* = 0.034, distance-threshold = 0.05) (Supplementary Fig. 5). When including all subtypes, all estimates were non-significant (Supplementary Fig. 6).

### Neurocognitive domains and PNP

In a sensitivity analysis, we determined the association between NDZS and diagnosis of PNP. Of the 981 PLWH with at least one visit, 28 were diagnosed with PNP. PNP was associated with lower sensory (*P* = 0.01) and motor skills (*P* = 0.0043) (Supplementary Fig. 7).

## Discussion

We showed here that neurocognitive performances correlate with HIV-1 residue variants, which we reported previously as associated with SRNC.^16^ Similarly, we determined a significant viral heritability of NDZS. While previous studies considered proxy or composite scores for HIV-1-associated NCIs, this is, to our knowledge, the first study to determine associations of performance among specific neurocognitive domains directly with HIV-1 residue variants.^16,17^

### SRNC validation

Given previous knowledge of viral heritability observed with SRNC (subjective data), we aimed to test whether the respective residues are also associated with NDZS (objective data); however, this was not the case.^16^ Nevertheless, the observed residue effect estimates for NDZS correlated significantly with the respective estimates for SRNC. This means that HIV-1 residues associated with increased SRNC (i.e. leading to a worse subjective outcome) were also associated with a NDZS reduction (i.e. leading to a worse objective outcome), and vice versa. We observed this across all NDZS, but particularly for sensory skills and speed of processing. Foremost, this validates the GWAS analysis of our previous study.^16^ In the case of NDZS showing no correlation, for example, executive functions and attention/working memory, these results cannot be seen as evidence for the absence of associated residues, just that they correlate poorly with SRNC.

We observed an increase in attention/working memory with Env N300G. This aligns with a previous report linking residue variants 300S and 300N to higher global deficit scores (GDS) (indicating increased NCI) compared to 300G.^18^ Notably, we observed increased attention/working memory (reflecting decreased NCI) with 300S, although without statistical significance. We observed similar patterns with Env Q328K and Q344E. While these variants were previously associated with HIV-associated dementia, our findings paradoxically showed improvements across neurocognitive domains.^41^ A possible explanation might be an absence of synergistic mutations within the three-dimensional protein structure to confer neurovirulence.^41^ Alternatively, differences in immune status may play a role. Our cohort was relatively healthy compared to PLWH with HIV-associated dementia, who are severely immunosuppressed.^41^ Selection pressure on the virus differs in these contexts, and similar residue variants may arise to serve distinct functions. For example, Q328K has been shown to decrease viral fitness.^42^ We can only speculate whether this reduced fitness limits neuroinvasion, thereby improving neurocognitive domains. Residue 344Q has been reported to play a role in immune evasion, and selective pressure on this site may indicate a healthier immune status, potentially associated with improvements in neurocognitive domains.^43^ Nevertheless, these observed effects could just arise by chance given the low statistical power.

We observed increased attention/working memory with Env E293K. While it was reported to favour CSF tropism, it was not observed to increase GDS.^44^ This indicates that while the residue variant is beneficial for the virus in the CSF, it does not increase its neurovirulence, consistent with our observed effect of increased attention/working memory.

We observed a trend for an increase in speed of processing with Env H308R. Residue 308R was previously found predominantly in plasma-derived virus, while 308H was predominantly found in CSF.^44^ The existing evidence that CSF tropism of 308H leads to increased neurovirulence is mixed.^44,45^ Nevertheless, our finding of increased speed of processing in the absence of 308H would support this hypothesis.

### Heritability

Some of the NDZS that correlated with SRNC, i.e. sensory and motor skills particularly, also show significant heritability. This is unsurprising, as both neurocognitive domains are linked to PNP, which is the most common HIV-1-associated neurological disorder in the ART era.^46^ We confirmed this in a sensitivity analysis, where PNP diagnosis was associated specifically with lower sensory and motor skills. Combined, this suggests that virus genetics are strongly associated with neurocognitive performance and could lead to NCI.

### Biological mechanisms and clinical implications

Our study allows us only to observe associations without causal inference. Nevertheless, research on other neurotropic viruses has shown diverse NCIs. While most seem to affect primarily executive functions, which as a complex higher-order domain is likely influenced by multiple pathogenic mechanisms, hepatitis B was additionally associated with impairments in language, hepatitis C with attention/working memory impairments and long COVID with impairments in attention/working memory and processing speed.^47-49^ These patterns may reflect the distinct cell compositions underlying cerebral structures involved in neurocognitive domains, with unique regulation of cellular pathways, receptor-, metabolite- and cytokine expression.^50,51^ For example, gp120, but not Nef or Tat, was shown to induce CXCR4-dependent endoplasmic reticulum stress in neurons.^52^ In contrast, Nef was shown to trigger an inflammatory CCL2 response in rat astrocytes, leading to memory impairments.^53^ Taken together, existing evidence points to multiple plausible mechanistic pathways, potentially exacerbated by viral mutations. In consequence, the clinical implications of our findings are uncertain. However, knowing the specific viral variants will help identify PLWH at highest risk for NCIs. While domain-specific neurorehabilitation strategies are under investigation, PLWH at highest risk may also benefit from domain-specific preventive approaches as well.^54^

### Limitations

The lack of significant residue variants, despite similar effect sizes, is likely caused by our limited sample size of HIV-1 WGS, especially for low-frequency residue variants and small effect sizes. HIV-1 WGS was restricted by sample availability in the biobank. For this reason, we restricted analyses to a validation of known residues instead of a full GWAS. The limited sample size may also reduce generalizability to the whole population. In a previous study, we showed that White, MSM, and HIV subtype B were overrepresented in PLWH with WGS data.^55^ Additionally, small sample sizes increase the probability of extreme observations. Furthermore, the high polygenicity of the phenotype further limits statistical power to identify relevant variants and their potential epistatic interactions. Therefore, the confirmed residue variants only account for a fraction of the genetic effects. While we adjusted for most risk factors and potential confounders, we cannot exclude the influence of unmeasured confounding, for example, host genetics, socioeconomic status and ART adherence. Additionally, due to the limited sample size, we were unable to adjust for all potential confounders, for example, CD4 nadir, ART regimens or duration of viral suppression. Moreover, we dichotomized several covariables to account for skewed distributions, which may have led to a loss of information and reduced confounder adjustment. While an AUC definition of the phenotype ensured comparability with prior studies, a longitudinal mixed-effect model may have been able to better capture non-linear effects.

## Conclusion

The present study validates HIV-1 residue variants we previously identified as SRNC associated. Notably, we show for the first time, to our knowledge, that performance among specific neurocognitive domains is differentially associated with the HIV-1 genome sequence. While these molecular signatures require validation in larger, multicentre cohorts and functional studies, they represent a meaningful step towards viral-guided neuro-prognosis. Further research and therapeutic approaches may benefit from targeting specific neurocognitive domains, such as motor and sensory skills.^54^ Ultimately, HIV-sequencing should be integrated with individual patient characteristics to support a personalized medicine framework, enabling clinicians to predict risk and implement domain-specific preventive strategies.

## Supplementary Material

fcag371_Supplementary_Data

## Data Availability

The individual-level datasets generated or analysed during the current study do not fulfil the requirements for open data access: (1) the SHCS informed consent states that sharing data outside the SHCS network is only permitted for specific studies on HIV infection and its complications, and to researchers who have signed an agreement detailing the use of the data and biological samples; and (2) the data are too dense and comprehensive to preserve patient privacy in PLWH. According to the Swiss law, data cannot be shared if data subjects have not agreed or the data are too sensitive to share. Investigators with a request for selected data should send a proposal to the respective SHCS address (www.shcs.ch/contact). The provision of data will be considered by the Scientific Board of the SHCS and the study team and is subject to Swiss legal and ethical regulations, and is outlined in a material and data transfer agreement. The underlying code is available at https://github.com/M-Zeeb/gwas_namaco.

## Appendix

**The Swiss HIV Cohort Study:** Irene A. Abela, Karoline Aebi-Popp, Alexia Anagnostopoulos, Manuel Battegay, Enos Bernasconi, Dominique L. Braun, Heiner C. Bucher, Alexandra Calmy, Matthias Cavassini, Angela Ciuffi, Günter Dollenmaier, Matthias Egger, Luisa Elzi, Jan S. Fehr, Jacques Fellay, Hansjakob Furrer, Christoph A. Fux, Huldrych F. Günthard, Anna Hachfeld, David H. Haerry, Barbara Hasse, Hans H. Hirsch, Matthias Hoffmann, Irene Hösli, Michael Huber, David Jackson-Perry, Christian R. Kahlert, Olivia Keiser, Thomas Klimkait, Roger D. Kouyos, Helen Kovari, Katharina Kusejko, Niklaus D. Labhardt, Karoline Leuzinger, de T. B. Martinez, Catja Marzolini, Karin J. Metzner, Nicolas Müller, Johannes Nemeth, Dunja Nicca, Julia Notter, Paolo Paioni, Giuseppe Pantaleo, Matthieu Perreau, Andri Rauch, Luisa P. Salazar-Vizcaya, Patrick Schmid, Olivier Segeral, Roberto F. Speck, Marcel Stöckle, Philip E. Tarr, Alexandra Trkola, Gilles Wandeler, Maja Weisser and Sabine Yerly.

**The NAMACO study group:** Matthias Cavassini, Renaud Du Pasquier, Mélanie Métral, Samanta Simioni, Peter Brugger, Klemens Gutbrod, Andreas U. Monsch, Ursi Kunze, Marianne Schneitter, Isaure Nadin, Severin Früh, Marc Schwind, Riccardo Pignatti, Stefanie Clarke, Frédéric Assal, Tobias Derfuss, Sebastian von Arx, Günter Eisele, Leonardo Sacco, Manuel Bertschi, Thomas Hundsberger, Alexandra Calmy, Thanh D. Lecompte, Christoph Hauser, Alexia Cusini, Rainer Weber, Helen Kovari, Barbara Hasse, Philip Tarr, Marcel Stöckle, Christoph Fux, Enos Bernasconi, Caroline Di Benedetto, Alessandra Bruno, Patrick Schmid, Katharine Darling, Alexandra Scherrer, Katharina Kusejko, Sandra Chaudron, Yannick Vallet, Deolinda Alves, Isabella Locatelli, Bruno Ledergerber, Laurent Decosterd, Catia Marzolini, Cristina Granziera, Gunnar Krueger, Reto Meuli and Maria Vargas.
