# Supplementary material for: Viral heritability of neurocognitive performances in persons living with HIV and underlying viral residue variants
Source: Brain Commun. 2026 Oct 5;8(5):fcag371. doi: 10.1093/braincomms/fcag371 (PMC13685630; doi:10.1093/braincomms/fcag371)
Supplement: Supplementary file 1 [file fcag371_supplementary_data.docx]

# **Supplementary material**

**Supplementary Figure 1** **No significant associations of variant residues with any of the seven neurocognitive domains for the residues previously identified and associated with self-reported neurocognitive complaints.**^1^ Each triangle represents a -log10 p value, corresponding to the association of the residue with the respective neurocognitive domain, obtained from an adjusted linear regression (N=192). The residue is indicated on the x axis, from the top: first the HIV gene, second the position, third the wildtype residue (used as a reference), and last the variant residue. Adjusted for multiple testing with the Benjamini-Hochberg procedure. Line at the top indicates the significance threshold.

**Supplementary Figure 2 Viral heritability among the seven neurocognitive domains.** **(A/B)** Phylogenetic trees based on partial pol sequences of all subtypes, including clustering sequences using a distance of 0.06 (N=647) **(A)** and 0.03 (N=227) **(B)**. Phylogenetic trees, including 1000 bootstraps, were inferred with IQ-Tree 2.2.0 using ModelFinder to select the substitution model. **(C)** Heritability estimates and their bootstrapped 95% confidence intervals, inferred with linear mixed effect models, incorporating phylogenetic clusters based on partial pol sequences (N_0.06_=647, N_0.05_=502, N_0.04_=350, N_0.03_=227) as random effects. Multivariable models were adjusted for risk factors and potential confounders.

**Supplementary Figure 3 Viral heritability among the seven neurocognitive domains.** Heritability estimates and their bootstrapped 95% confidence intervals, inferred with partial pol sequences (N=981) and an Ornstein-Uhlenbeck model (OU) and Brownian motion model (BM). Multivariable models were adjusted for risk factors and potential confounders.

**Supplementary Figure 4 Viral heritability among the seven neurocognitive domains.** Heritability estimates and their bootstrapped 95% confidence intervals, inferred with whole genome sequences (N=192) and an Ornstein-Uhlenbeck model (OU) and Brownian motion model (BM). Multivariable models were adjusted for risk factors and potential confounders.

**Supplementary Figure 5 Viral heritability over the evolution of cognitive performances (considering three visits) among the seven neurocognitive domains.** **(A/B)** Phylogenetic trees based on partial pol sequences of subtype B, including clustering sequences using a distance of 0.06 (N=284) **(A)** and 0.05 (N=219) **(B)**. Phylogenetic trees, including 1000 bootstraps, were inferred with IQ-Tree 2.2.0 using ModelFinder to select the substitution model. **(C)** Heritability estimates and their bootstrapped 95% confidence intervals, inferred with linear mixed effect models, incorporating phylogenetic clusters based on partial pol sequences (N_0.06_=284, N_0.05_=219, N_0.04_=149) **(A/B)** as random effects. Multivariable models were adjusted for risk factors and potential confounders.

**Supplementary Figure 6 Viral heritability over the evolution of cognitive performances (considering three visits) among the seven neurocognitive domains. (A/B)** Phylogenetic trees based on partial pol sequences of all subtypes, including clustering sequences using a distance of 0.06 (N=329) **(A)** and 0.05 (N=258) **(B)**. Phylogenetic trees, including 1000 bootstraps, were inferred with IQ-Tree 2.2.0 using ModelFinder to select the substitution model. **(C)** Heritability estimates and their bootstrapped 95% confidence intervals, inferred with linear mixed effect models, incorporating phylogenetic clusters based on partial pol sequences (N_0.06_=329, N_0.05_=258, N_0.04_=165) **(A/B)** as random effects. Multivariable models were adjusted for risk factors and potential confounders.

**Supplementary Figure 7 Global Z-scores for the seven neurocognitive domains** for persons living with HIV (PLWH) with vs without peripheral neuropathy (PNP) (N=981, N_PNP_=28). Each dot represents the Domain Z score of a PLWH at the first follow-up in the Neurocognitive Assessment in the Metabolic and Aging Cohort study (NAMACO). P values were derived with a Wilcoxon rank-sum test.

**Supplementary Table 1 Previously identified viral residues associated with self-reported neurocognitive complaints (SRNC)**^1^

| **Protein** | **Position** | **Wildtype Residue** | **Variant Residue** | **Present in Study** | **Associated with** |
| --- | --- | --- | --- | --- | --- |
| Env | 46 | K | R | TRUE | freq |
| Env | 291 | S | A | TRUE | conc |
| Env | 463 | N | S | TRUE | conc |
| Env | 464 | T | N | TRUE | conc  all; freq |
| Env | 641 | L | E | TRUE |  |
| Env | 683 | K | R | TRUE | all  slow  conc  all; slow; conc; freq |
| Gag | 252 | N | G | TRUE |  |
| Gag | 372 | N | G | TRUE |  |
| Gag | 387 | R | K | TRUE |  |
| Gag | 441 | H | S | TRUE | slow |
| Pol | 9 | P | Q | TRUE | slow |
| Pol | 332 | D | E | TRUE | freq; all  conc |
| Pol | 590 | V | I | TRUE |  |
| Rev | 18 | L | I | TRUE | all; freq; slow; conc |
| Rev | 100 | P | S | TRUE | freq  freq; conc; slow; all  slow  slow; conc |
| Tat | 64 | T | N | TRUE |  |
| Tat | 101 | D | L | TRUE |  |
| Vif | 155 | T | A | TRUE |  |
| Vif | 181 | K | R | TRUE | slow |
| Env | 19 | I | T | FALSE | slow |
| Env | 134 | L | W | FALSE | slow; conc |
| Env | 149 | M | S | FALSE | all |
| Env | 187 | D | K | FALSE | freq |
| Env | 190 | S | N | FALSE | freq |
| Env | 275 | E | A | FALSE | conc  freq |
| Env | 534 | S | A | FALSE |  |
| Gag | 232 | R | K | FALSE | slow; all  conc; all  slow  slow |
| Gag | 373 | S | A | FALSE |  |
| Gag | 441 | H | N | FALSE |  |
| Nef | 8 | S | N | FALSE |  |
| Pol | 646 | L | P | FALSE | conc; all; slow  freq; all |
| Rev | 14 | K | T | FALSE |  |
| Rev | 91 | T | N | FALSE | conc; all; slow |
| Vif | 63 | K | E | FALSE | slow |
| Vif | 134 | E | N | FALSE | conc |
| Vpr | 37 | P | L | FALSE | all; slow |

Abbreviations: conc (concentration difficulties); freq (frequent memory loss); slow (cognitive slowing); all (combination of conc, freq, and slow).

**Supplementary Table 2 Previously reported viral variant residues**

| **Protein** | **Position** | **Present in Study** | **Published in** | **Associated with** |
| --- | --- | --- | --- | --- |
| Env | 283 | TRUE | ^2^ | HAND |
| Env | 287 | TRUE | ^2^ | HAND |
| Env | 290 | TRUE | ^2^ | HAND |
| Env | 291 | TRUE | ^2,3^ | HAND |
| Env | 293 | TRUE | ^4^ | CSF tropism |
| Env | 297 | TRUE | ^2^ | HAND |
| Env | 300 | TRUE | ^5^ | Global deficit score |
| Env | 307 | TRUE | ^2^ | HAND |
| Env | 308 | TRUE | ^2,4–6^ | CSF tropism; HAND; Global deficit score |
| Env | 315 | TRUE | ^2,3^ | HAND |
| Env | 318 | TRUE | ^2^ | HAND |
| Env | 319 | TRUE | ^2^ | HAND |
| Env | 328 | TRUE | ^2^ | HAND |
| Env | 334 | TRUE | ^2^ | HAND |
| Env | 340 | TRUE | ^3^ | HAND |
| Env | 343 | TRUE | ^2^ | HAND |
| Env | 344 | TRUE | ^2^ | HAND |
| Env | 317 | FALSE | ^2^ | HAND |
| Env | 321 | FALSE | ^2^ | HAND |
| Env | 332 | FALSE | ^6^ | HAND |
| Env | 339 | FALSE | ^2^ | HAND |
| Env | 341 | FALSE | ^4^ | CSF tropism |

Abbreviations: HAND (HIV associated neurocognitive disorder); CSF (cerebrospinal fluid).

**Supplementary Table 3 Origin of NGS sequences for SRNC validation**

| **Protein** | **Plasma RNA** | **Proviral DNA** | **Proportion Proviral** |
| --- | --- | --- | --- |
| Env | 69 | 104 | 60% |
| Gag | 71 | 113 | 61% |
| Nef | 65 | 110 | 63% |
| Pol | 72 | 117 | 62% |
| Rev | 68 | 98 | 59% |
| Tat | 68 | 98 | 59% |
| Vif | 64 | 102 | 61% |
| Vpr | 63 | 98 | 61% |
| Vpu | 68 | 97 | 59% |

**Supplementary Table 4 Population characteristics stratified by GWAS availability**

|  | **GWAS not available** | **GWAS available** |
| --- | --- | --- |
| **Characteristics* at NAMACO Follow Up** | **N = 359** | **N = 192** |
| Neurocognitive Domain Global Z scores |  |  |
| Verbal episodic memory | 0.22 (-0.33, 0.78) | 0.22 (-0.45, 0.89) |
| Attention/Working memory | 0.17 (-0.34, 0.67) | -0.09 (-0.50, 0.50) |
| Speed of processing | 0.35 (-0.21, 0.83) | 0.07 (-0.51, 0.55) |
| Executive functions | 0.34 (-0.06, 0.65) | 0.27 (-0.03, 0.58) |
| Language abilities | 0.64 (-0.06, 1.20) | 0.31 (-0.33, 0.87) |
| Motor skills | -0.17 (-0.67, 0.34) | -0.25 (-0.83, 0.33) |
| Sensory skills | 0.34 (-0.21, 0.94) | 0.23 (-0.30, 0.90) |
| Birth year | 1,961 (1,956, 1,964) | 1,962 (1,956, 1,965) |
| Age [years] | 58 (54, 62) | 56 (53, 63) |
| Sex [female] | 72 (20%) | 36 (19%) |
| Higher Education completed | 314 (87%) | 172 (90%) |
| HIV Transmission mode |  |  |
| MSM | 200 (56%) | 110 (57%) |
| HET | 108 (30%) | 61 (32%) |
| Other | 51 (14%) | 21 (11%) |
| Ethnicity Non-White | 27 (7.5%) | 19 (9.9%) |
| EFV use | 209 (58%) | 122 (64%) |
| Antidepressant use | 113 (31%) | 56 (29%) |
| Depression | 172 (48%) | 85 (44%) |
| Recreational drug use | 50 (14%) | 29 (15%) |
| Neurological disease/event | 38 (11%) | 25 (13%) |
| Hep C | 53 (15%) | 34 (18%) |
| Hep B | 101 (28%) | 61 (32%) |

* median (interquartile range) / n (%)

# **Supplementary material references**

1.	Zeeb M, Pasin C, Cavassini M, et al. Self-reported neurocognitive complaints in the Swiss HIV Cohort Study - a viral genome wide association study. *Brain Commun*. Published online May 31, 2024. doi:10.1093/BRAINCOMMS/FCAE188

2.	Holman A, Gabuzda D. A Machine Learning Approach for Identifying Amino Acid Signatures in the HIV Env Gene Predictive of Dementia. *PLoS One*. 2012;7:e49538. doi:10.1371/journal.pone.0049538

3.	Ogishi M, Yotsuyanagi H. Prediction of HIV-associated neurocognitive disorder (HAND) from three genetic features of envelope gp120 glycoprotein. *Retrovirology*. 2018;15(1):12. doi:10.1186/s12977-018-0401-x

4.	Strain MC, Letendre S, Pillai SK, et al. Genetic composition of human immunodeficiency virus type 1 in cerebrospinal fluid  and blood without treatment and during failing antiretroviral therapy. *J Virol*. 2005;79(3):1772-1788. doi:10.1128/JVI.79.3.1772-1788.2005

5.	Pillai SK, Pond SLK, Liu Y, et al. Genetic attributes of cerebrospinal fluid-derived HIV-1 env. *Brain*. 2006;129(7):1872-1883. doi:10.1093/brain/awl136

6.	Power C, McArthur JC, Johnson RT, et al. Demented and nondemented patients with AIDS differ in brain-derived human immunodeficiency virus type 1 envelope sequences. *J Virol*. 1994;68(7):4643-4649. doi:10.1128/jvi.68.7.4643-4649.1994
